# Atrial Fibrillation Recurrence and Peri-Procedural Complication Rates in nMARQ vs. Conventional Ablation Techniques: A Systematic Review and Meta-Analysis

**DOI:** 10.3389/fphys.2018.00544

**Published:** 2018-05-22

**Authors:** Ka H. C. Li, Mei Dong, Mengqi Gong, George Bazoukis, Ishan Lakhani, Yan Y. Ting, Sunny H. Wong, Guangping Li, William K. K. Wu, Vassilios S. Vassiliou, Martin C. S. Wong, Konstantinos Letsas, Yimei Du, Victoria Laxton, Bryan P. Yan, Yat S. Chan, Yunlong Xia, Tong Liu, Gary Tse, International Health Informatics Study (IHIS) Network

**Affiliations:** ^1^Faculty of Medicine, Newcastle University, Newcastle upon Tyne, United Kingdom; ^2^Department of Medicine and Therapeutics, Faculty of Medicine, Chinese University of Hong Kong, Hong Kong, China; ^3^Li Ka Shing Institute of Health Sciences, Faculty of Medicine, Chinese University of Hong Kong, Hong Kong, China; ^4^Department of Cardiology, The Affiliated Yantai Yuhuangding Hospital of Qingdao University, Yantai City, China; ^5^Tianjin Key Laboratory of Ionic-Molecular Function of Cardiovascular Disease, Department of Cardiology, Tianjin Institute of Cardiology, Second Hospital of Tianjin Medical University, Tianjin, China; ^6^Laboratory of Cardiac Electrophysiology, Second Department of Cardiology, Evangelismos General Hospital of Athens, Athens, Greece; ^7^Key Laboratory of Cardiovascular Remodeling and Function Research, Chinese Ministry of Education and Chinese Ministry of Health, Department of Cardiology, Shandong University Qilu Hospital, Jinan, China; ^8^Department of Anaesthesia and Intensive Care, State Key Laboratory of Digestive Disease, LKS Institute of Health Sciences, The Chinese University of Hong Kong, Hong Kong, China; ^9^Norwich Medical School, University of East Anglia, Norwich, United Kingdom; ^10^The Jockey Club School of Public Health and Primary Care, Faculty of Medicine, The Chinese University of Hong Kong, Hong Kong, China; ^11^Research Center of Ion Channelopathy, Institute of Cardiology, Union Hospital, Tongji Medical College, Huazhong University of Science and Technology, Wuhan, China; ^12^Department of Cardiology, First Affiliated Hospital of Dalian Medical University, Dalian, China

**Keywords:** nMARQ, nMARQ™, ablation, atrial fibrillation, recurrence

## Abstract

**Background and Objectives:** Atrial fibrillation is a common abnormal cardiac rhythm caused by disorganized electrical impulses. AF which is refractory to antiarrhythmic management is often treated with catheter ablation. Recently a novel ablation system (nMARQ) was introduced for PV isolation. However, there has not been a systematic review of its efficacy or safety compared to traditional ablation techniques. Therefore, we conducted this meta-analysis on the nMARQ ablation system.

**Methods:** PubMed and EMBASE were searched up until 1st of September 2017 for articles on nMARQ. A total of 136 studies were found, and after screening, 12 studies were included in this meta-analysis.

**Results:** Our meta-analysis shows that the use of nMARQ was associated with higher odds of AF non-recurrence (*n* = 1123, odds ratio = 6.79, 95% confidence interval 4.01–11.50; *P* < 0.05; I^2^ took a value of 83%). Moreover, the recurrence rate of AF using nMARQ was not significantly different from that of traditional ablation procedures (*n* = 158 vs. 196; *OR* = 0.97, 95% confidence interval:0.59–1.61). No significant difference in complication rates was observed between these groups (RR: 0.86; 95% CI: 0.37–1.99; *P* > 0.05). There were four reported mortalities in the nMARQ group compared to none in the conventional ablation group (relative risk: 1.58; 95% CI: 0.09–29.24; *P* > 0.05).

**Conclusions:** AF recurrence rates are comparable between nMARQ and conventional ablation techniques. Although general complication rates are similar for both groups, the higher mortality with nMARQ suggests that conventional techniques should be used for resistant AF until improved safety profiles of nMARQ can be demonstrated.

## Introduction

Atrial fibrillation (AF) is the most common arrhythmia encountered in clinical practice. It can have both re-entrant and triggered mechanisms (Tse et al., 2016), the latter exemplified by impulses originating from the roots of the pulmonary veins (Hu et al., 2015). One of the major concerns associated with AF is an increased risk of thrombo-embolic events (stroke or systemic embolism). Anticoagulation therapies are therefore recommended in all patients with AF who are at moderate-to-high risk of stroke (Singer et al., 2008; Camm et al., 2010), which include the presence of co-morbidities such as type 2 diabetes mellitus (Marfella et al., 2013; Steinberg et al., 2015). As well as the increased risk of thrombo-embolic events, AF also remains a major aetiological factor of heart failure and increased hospitalization rates. As such, establishing an effective monitoring system for early AF detection along with an effective approach to treating AF is essential (Sardu et al., 2016).

Numerous studies have demonstrated the superiority of interventions over pharmacological approaches for the maintenance of sinus rhythm in patients with both paroxysmal and persistent AF. Considering that the pulmonary vein (PV) can produce rapid focal activation that contributes to AF persistence, disruption of the electrical connection between the left atrium and the left and right PVs by circumferential PV isolation may prevent occurrence of the arrhythmia (Calkins et al., 2012b; Camm et al., 2012). Apart from the irrigated single-tip, point-by-point delivery technique, innovative technologies such as single-shot devices, balloon technology, and circumferential multipolar ablation catheters have been introduced over the last decade as alternatives for ablation procedures. These new ablation tools have allowed for safer and more efficient isolation by applying different forms of energy to create linear lesions at the peri-PV ostia region (Deneke et al., 2011; Schade et al., 2012; Packer et al., 2013).

Recently, circular irrigated radiofrequency ablation using the novel ablation system, nMARQ, (Biosense Webster, Diamond Bar, CA, USA) has been introduced for circumferential PV isolation. Several studies have compared nMARQ with conventional ablation tools. However, the definite efficacy of this new system has not been clearly elucidated due to differing results from the studies and there has not been a systematic evaluation to date. In this study, we therefore conducted a systematic review and meta-analysis to examine AF recurrence as well as peri-procedural complications between the nMARQ ablation system and traditional ablation techniques.

### Pathophysiology of AF

Currently, a combination of triggered and re-entrant mechanisms involving not only the atrium itself but structures such as ganglionated plexi and the pulmonary veins have been proposed to underlie the generation and maintenance of AF (Calkins et al., 2012a). Autonomic modulation is thought to be an important mediator of arrhythmogenesis (Marrouche et al., 2014; Rizzo et al., 2015). Recently, Yang Felix et al. proposed a common pathophysiological pathway that can cause the development and progression of AF associated with inflammatory and fibrotic changes (Yang et al., 2017). This was supported by Cochet et al. who described the difference in atrial fibrosis distribution between patients with and without AF (Cochet et al., 2015).

## Methods

### Search strategy, inclusion, and exclusion criteria

The meta-analysis was performed according to the Preferred Reporting Items for Systematic Reviews and Meta-Analyses statement (Moher et al., 2009). PubMed and EMBASE were searched for studies that investigated AF recurrence rates using nMARQ and/or conventional ablation techniques. The following terms were used: “nMARQ” and “nMARQ™.” The search period was from the beginning of the databases through to 1st September 2017 with no language restrictions. The following inclusion criteria were applied: (i) the design was a case-control, prospective or retrospective cohort study in humans, (ii) AF recurrence and complication rates were reported for nMARQ with or without comparison to conventional ablation techniques. Included studies also adhered to the follow-up recommendations post-ablation from the 2016 ESC guidelines for the management of atrial fibrillation developed in collaboration with EACTS. These suggest that “patients should be seen at least once by a rhythm specialist in the first 12 months after ablation” (Kirchhof et al., 2016).

The quality assessment of these studies included in our meta-analysis was performed using the Newcastle–Ottawa Quality Assessment Scale (NOS). The point score system evaluated the categories of study participant selection, comparability of the results, and quality of the outcomes. The following characteristics were assessed: (a) representativeness of the exposed cohort; (b) selection of the non-exposed cohort; (c) ascertainment of exposure; (d) demonstration that outcome of interest was not present at the start of study; (e) comparability of cohorts on the basis of the design or analysis; (f) assessment of outcomes; (g) follow-up period sufficiently long for outcomes to occur; and (h) adequacy of follow-up of cohorts. This scale varied from zero to nine stars, which indicated that studies were graded as poor quality if they met <5 criteria, fair if they met 5 to 7 criteria, and good if they met >8 criteria. The details of the NOS quality assessment are shown in Supplementary Table 1.

### Data extraction and statistical analysis

Data from the studies were entered in a pre-specified spreadsheet in Microsoft Excel. All publications identified were assessed for compliance with the inclusion criteria. In this meta-analysis the extracted data elements consisted of: (i) publication details: surname name of first author, publication year; (ii) study design; (iii) follow-up duration; (iv) the quality score; and (v) the characteristics of the population including sample size, gender, age. Two reviewers (CL and MD) independently reviewed each included study and disagreements were resolved by adjudication with input from a third reviewer (TL). Research findings from abstracts are frequently significantly different from the final publication and have not undergone the same degree of rigorous peer review process as normally required for journal articles. For these reasons only full-text publications were included in this meta-analysis.

Heterogeneity across studies was determined using Cochran's *Q*-value and the *I*^2^ statistic from the standard chi-square test. Cochran's *Q*-value is the weighted sum of squared differences between individual study effects and the pooled effect across studies. The *I*^2^ statistic from the standard chi-square test describes the percentage of variability in the effect estimates resulting from heterogeneity. *I*^2^ > 50% was considered to reflect significant statistical heterogeneity. The random-effects model using the inverse variance heterogeneity method was used with *I*^2^ > 50%. To locate the origin of the heterogeneity, subgroup analyses based on different disease conditions and different endpoints were performed. Sensitivity analysis excluding one study at a time was also performed. Funnel plots showing standard errors or precision against the logarithms of the odds ratio were constructed. The Begg and Mazumdar rank correlation test and Egger's test were used to assess for possible publication bias.

## Results

### Efficacy of the nMARQ ablation technique

**Figure 1:**
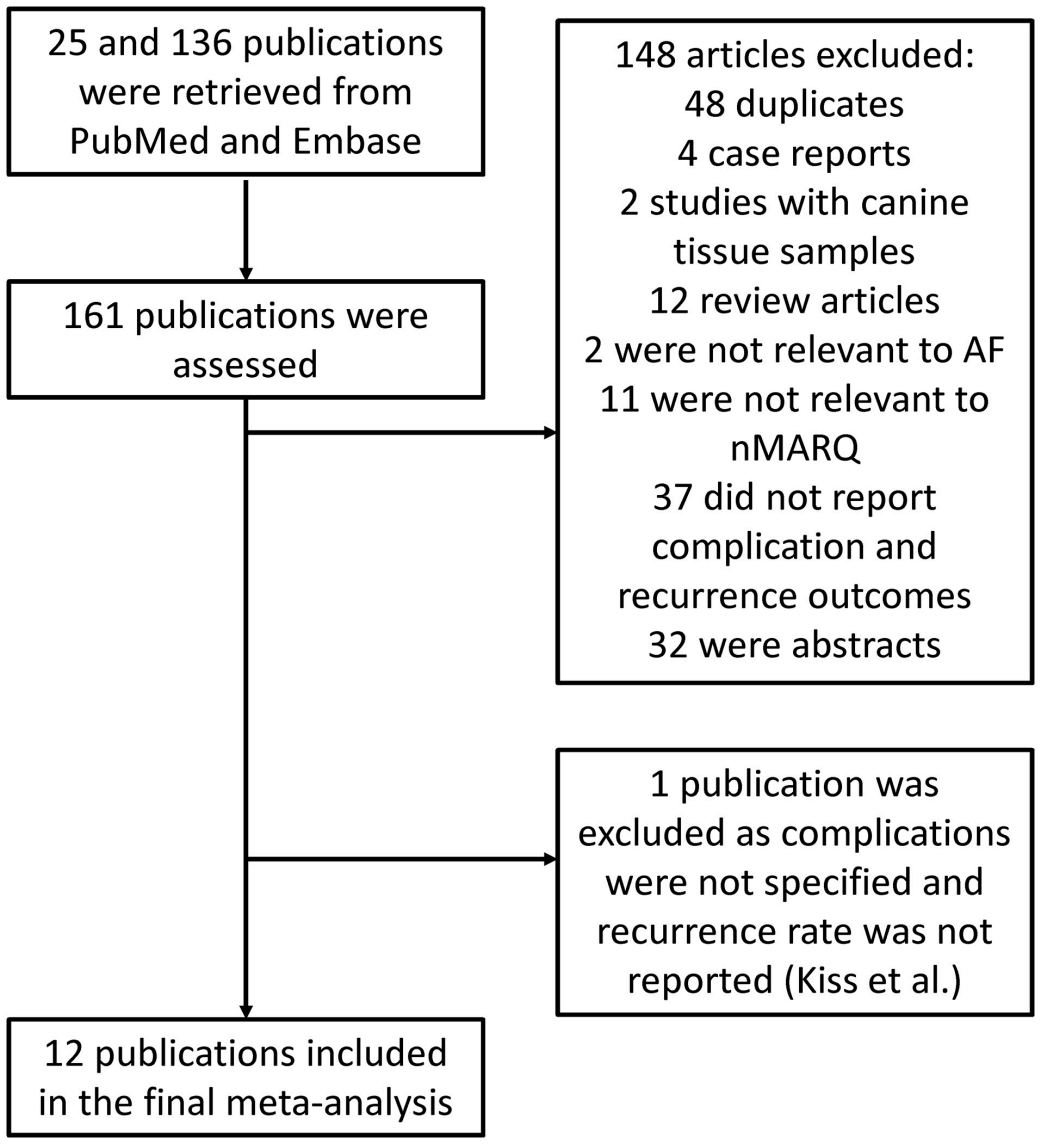
Flowchart of the database search and study selection process.

A flow diagram detailing the above search strategy with inclusion and exclusion criteria is shown in Figure 1. A total of 31 publications were found and further assessment demonstrated that 13 met the inclusion criteria. The Kiss et al. (2014) study was excluded due to AF recurrence not being reported as an endpoint. Therefore, a total of 12 studies were included in this meta-analysis (Scaglione et al., 2014; Zellerhoff et al., 2014; Dello Russo et al., 2015; Farkash et al., 2015; Mahida et al., 2015; Burri et al., 2016; Laish-Farkash et al., 2016; Lauschke et al., 2016; Rodriguez-Entem et al., 2016; Rosso et al., 2016; Vurma et al., 2016; Wakili et al., 2016). Of these, six reported efficacy of the novel nMARQ ablation system without any comparison. Four studies compared it to other ablation techniques such as single-catheter ablation catheterisation (SAC) (Lauschke et al., 2016; Wakili et al., 2016), “Smart Touch” radiofrequency (Rosso et al., 2016) and pulmonary vein ablation catheterisation (Laish-Farkash et al., 2016) which uses two circular multi-electrode catheters. Two studies divided the nMARQ sample into either different technical approaches (Dello Russo et al., 2015) or into the efficacy of nMARQ in paroxysmal and persistent AF (Vurma et al., 2016). The baseline characteristics of these studies are listed in Table 1. Three were retrospective studies and nine were prospective studies. The mean follow-up duration was 9.3 months based on 11 out of 12 studies as one study did not provide information regarding this.

**Table 1:** Baseline characteristics of the included studies.

	**Sample no. (n)**	**Mean age (years ± SD)**	**Males n(%)**	**Paroxysmal AF n(%)**	**Mean LVEF (%± SD)**	**Mean Follow-up (Months)**	**Total procedure time (min ± SD)**	**Fluoroscopy time (min ± SD)**	**Cum. Radiofrequency time (min ± SD)**	**Acute success rate n(%)**	**Periprocedural complications (n)**
Burri et al., 2016	50	58 ± 10	37(74)	50(100)	55 ± 5*$*	15	100 ± 25	22 ± 8	13.3 ± 2.2	–(–)	2
Dello Russo et al., 2015	37	53.5 ± 10	28(75.5)	31(83.8)	61 ± 3.5	3			−	–(–)	0
							nMARQ with ICE	nMARQ with ICE			
							83 ± 23	23.1 ± 9			
							nMARQ w/ fluoroscopy + TC tech	nMARQ w/ fluoroscopy + TC tech			
							160 ± 42	27.7 ± 5			
Laish-Farkash et al., 2016	82	63 ± 10.6	55(67)	62(76)	–(–)	> 5	81 ± 18	30 ± 8.5	−	78(95)	8
Lauschke et al., 2016	11	59.6 ± 8	8(72.7)	6(54.5)	53 ± 15	10.6	111.8 ± 34.9	20.5 ± 8.9	6.3 ± 3	–(–)	1
Mahida et al., 2015	374	60 ± 10	264(70.6)	263(70.3)	60 ± 8	12	114 ± 42	24 ± 14	13.5	243(65)	2
Rodriguez-Entem et al., 2016	35	57.3 ± 8.6	28(80)	35(100)	62.6 ± 5.8	16.8	79.5 ± 39.3	31.6 ± 8.2	7.9	33(94.2)	1
Rosso et al., 2016	36	58 ± 10	27(75)	23(64)	–(–)	20.3	101 ± 26.4	25.9 ± 9.5	–	28(78)	0
Scaglione et al., 2014	25	57 ± 13	19(76)	25(100)	61 ± 6	6	131 ± 49	1.8 ± 2	14.9 ± 3.7	24(96)	3
Vurma et al., 2016	327	Paroxysmal	131(40.1)	–(–)	Paroxysmal	6	Paroxysmal	Paroxysmal	Paroxysmal	−(−)	17
		63 ± 10			62.4 ± 10		68.6 ± 22.5	14.8 ± 6.6	18.9 ± 6.4		
		Persistent			Persistent		Persistent	Persistent	Persistent		
		64.8 ± 8.2			57.5 ± 11		75 ± 22.7	16.8 ± 6.3	22.1 ± 6.1		
Wakili et al., 2016	29	64.3 ± 11.1	16(55)	29(100)	61.5 ± 12.1	13.3	132 ± 37	31 ± 12	21 ± 9	−(−)	4
Zellerhoff et al., 2014	39	60 ± 10	31(79)	39(100)	65 ± 7	5	86 ± 29	22.2 ± 6.5	10 ± 4.6	37(95)	1
Farkash et al., 2015 (group 1)	37	64 ± 10.5	23(62)	32(86.5)	–(–)	12	78 ± 19	30 ± 9	10.3 ± 3.6	36(97)	7
Farkash et al., 2015 (group 2)	41	62.5 ± 11	29(71)	27(66)	–(–)	12	85.5 ± 18.5	29.5 ± 8.7	12 ± 4	40(97.5)	1

### Efficacy of the nMARQ ablation technique compared to conventional ablation techniques

**Figure 2:**
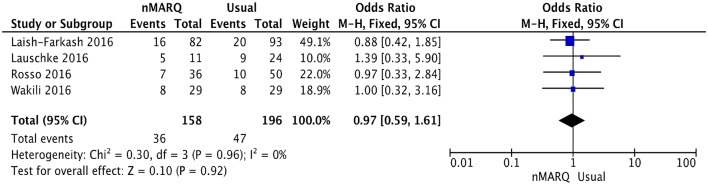
Efficacy of the nMARQ ablation technique compared to conventional ablation techniques.

The conventional ablation techniques include (i) “point-by-point” radiofrequency using a single irrigated tip ablation catheter and (ii) pulmonary vein ablation catheter, which uses two circular multi-electrode catheters. Three studies compared nMARQ with single-tip ablation catheter and one with the two circular multi-electrode catheters. A total of 158 patients were treated with nMARQ compared to 196 patients undergoing conventional ablation procedures (Table 2). The mean age for the conventional ablation group was 61.5 ± 10.5 years and 61.2% of the subjects were male. The mean total procedure time was 103.8 ± 32.4 min and the mean total fluoroscopy time was 27.9 ± 12.4 min. Our meta-analysis shows that the recurrence rate of AF using nMARQ was not significantly different from that of traditional ablation procedures (*OR* = 0.97, 95% confidence interval: 0.59–1.61; Figure 2).

**Table 2:** Efficacy of the nMARQ ablation technique compared to other techniques.

	**Sample no. (n)**	**Mean age (years ± SD)**	**Males n(%)**	**Paroxysmal AF n(%)**	**Mean LVEF (%± SD)**	**Mean Follow-up (Months)**	**Total procedure time (min ± SD)**	**Fluoroscopy time (min ± SD)**	**Cum. Radiofrequency time (min ± SD)**	**Acute success rate n(%)**	**Periprocedural complications (n)**
Wakili et al., 2016	29	64.3 ± 11.1	16(55)	29(100)	63.4 ± 7.1	13.3	109 ± 30	23 ± 10	35 ± 12	−	1
Rosso et al., 2016	50	62 ± 8	32(64)	34(68)	−	19.7	105 ± 16.6	24 ± 6	−	41(82)	0
Laish-Farkash et al., 2016	93	61 ± 10	56(60)	81(87)	−	>5	94 ± 27	33 ± 13	−	90(97)	5
Lauschke et al., 2016	24	59.2 ± 12.3	16(66.6)	20(83.3)	59 ± 8	13.6	132.7 ± 48.2	22.4 ± 9.4	18.6 ± 13.9	−	0

### Peri-procedural complications

All studies included in the analysis provided data on perioperative complications. A total of 47 peri-procedural complications (4.19%) occurred in the nMARQ group (*n* = 1123) while complications were observed in 6 patients (3.06%) in the conventional ablation group (*n* = 196). The following complications occurred following the use of nMARQ: groin hematomas (*n* = 17), transient ST-segment elevation (*n* = 8), access site injury (*n* = 8), death (*n* = 4), pericardial tamponade (*n* = 4), pericardial effusion (*n* = 3), phrenic nerve palsy (*n* = 1), oesophageal lesion (*n* = 1), charring injury (*n* = 1). Regarding the complications with the conventional ablation techniques the following occurred: transient ST-segment elevation (*n* = 3), access site injuries (*n* = 2), groin haematoma (*n* = 1). In terms of peri-procedural mortality, four were reported in the nMARQ group from the Mahida et al. and Vurma et al. studies (Mahida et al., 2015; Vurma et al., 2016). Three deaths were attributed to procedure-induced esophageal-pericardial fistulae. The remaining death was due to sepsis (Mahida et al., 2015). By contrast, there was zero mortality in the conventional ablation group.

Additionally, when head-to-head analysis was conducted, the conventional ablation group was associated with lower odds of periprocedural complications (Odds ratio: 2.59; 95% CI: 0.98–6.80; *P* = 0.05). In the head-to-head analysis, the nMARQ group had 1 phrenic nerve palsy, 1 oesophageal lesion, 1 groin haematoma, 1 charring injury, 1 pericardial tamponade, 1 pericardial effusion, 3 transient ST-elevations and 4 access site injuries. In the conventional ablation group there was only 1 haematoma, 3 transient ST-elevations, and 2 access site injuries.

## Discussion

This systematic review and meta-analysis evaluated the efficacy of the nMARQ catheter against conventional ablation approaches. Since the main objective of ablation is to treat drug-resistant AF, this study defined AF recurrence as the end-point. All studies adhered to the post-ablation follow-up 2016 ESC guidelines for the management of AF developed in collaboration with EACTS. Physical examinations, evaluation of symptoms, 12-lead ECG recordings, transthoracic echocardiography and Holter ECG recordings (ranging between 1 and 7 days) were included in follow-up monitoring. According to multiple studies included in this meta-analysis AF recurrence is assumed as any atrial tachyarrhythmia lasting at least 30 s on an ECG loop recorder or ECG, regardless if it is organized into flutter or not.

The main findings are that (i) the use of the nMARQ catheter is a useful technique in resolving treatment resistant AF accompanied by low rates of recurrences; ii) when cross-analyzed with conventional techniques, nMARQ is equally as effective as conventional ablation procedures; iii) overall periprocedural complication risk was greater with the use of nMARQ compared to conventional techniques, iv) when mortality was analyzed as a separate end-point higher mortality was observed in the nMARQ group but this did not achieve statistical significance.

### Procedural parameters

The mean total procedure time (94.6 ± 18.7 vs. 103.8 ± 32.4) and fluoroscopy time (22.1 ± 8.8 vs. 27.9 ± 12.4) were significantly shorter for nMARQ compared to conventional approaches. This difference is due to the variability in the transseptal and procedural approach. Some studies used a dual trans-septal access approach while others used a single-access approach without using a circular mapping catheter (CMC) to confirm pulmonary vein isolation. Studies that used PV mapping were found to have a longer fluoroscopy time of 31–35 min compared to the 20–24 min fluoroscopy time in studies without PV mapping (Wakili et al., 2016). Another possible difference in the procedure and fluoroscopy time is the “learning curve.” In Wakili et al. there was no observable trend in procedural parameters with time. However, in Rosso et al. a significant learning curve was observed with decreasing fluoroscopy and procedural times (Wakili et al., 2016). Burning time was shorter for nMARQ compared to PVAC. The longer total burning time was attributed to the availability of 3D mapping used with nMARQ and not with PVAC. Additionally, nMARQ ablation can be stopped at any point after PV signals are no longer detected after 1 min (Wakili et al., 2016). In terms of left ventricular ejection fraction (LVEF), the values were similar between the nMARQ group (60.4 ± 10.3) and the “Usual” group, which were only reported specifically by Wakili et al. and Lauschke et al. as 63.4 ± 7.1 and 59 ± 8 respectively.

### Advantages of nMARQ over conventional ablation techniques

The availability of 3D mapping with nMARQ confers many advantages over conventional ablation techniques. It allows visualization of catheter position in relation to the PV ostia, guides voltage mapping of the atrium and adds location points of the phrenic nerve route. Moreover, fluoroscopy time can be reduced by using CARTO-MERGE technology. Lines of ablations outside PV ostium can also be added. According to the same study higher atrial arrhythmia incidence was observed for PVAC when compared to nMARQ patients (95 vs. 36.5%, *P* = 0.0001). The origin of the arrhythmogenic activity with PVAC system can be due to the presence of a guide wire stimulating the PV ostia or the different energy used with unipolar electrodes in nMARQ compared to bipolar electrodes in PVAC (Wakili et al., 2016).

### Concerns with success in achieving pulmonary vein isolation and peri-procedural complications in nMARQ vs. conventional ablation procedures

Confirming ablation success is impeditive for accurately predicting AF recurrence. This is because an incomplete PVI will more likely give rise to a post-procedural AF. The use of the novel circulation ablation catheter, nMARQ, has raised concerns with regard to its ability to successfully achieve successful PVI (von Bary et al., 2011; Wakili et al., 2016). However, Scaglione et al. and Rosso et al. have adequately addressed this issue (Rosso et al., 2014; Scaglione et al., 2014). Rosso et al. suggested that the nMARQ catheter is associated with poor signal concordance after radiofrequency (RF) application. Their group used a dual transseptal approach instead of the single approach from the start of the procedure, thereby potentially facilitating a successful ablation of residual PV conduction by simultaneous PV mapping with the CMC. The nMARQ most commonly missed persistent atrial PV conduction which was observed in 30% of the examined PVs using a CMC. Similarly, in Scaglione et al., 22% of PVs were found to be persisting post-procedure even though the nMARQ catheter suggested complete PVI. These findings are in keeping with the many studies on nMARQ alone that have all indicated >98% of PVIs are successful (Scaglione et al., 2014; Zellerhoff et al., 2014; Mahida et al., 2015; Burri et al., 2016; Rodriguez-Entem et al., 2016). This is an important aspect to address as it will determine if nMARQ could effectively substitute existing approaches or if supplementary post-RF conduction is required to confirm ablation success (Wakili et al., 2016). Indeed, Lauschke et al. confirmed that complete PV re-isolation is possible with nMARQ (Lauschke et al., 2016). The difficulty in sufficiently isolating the left-inferior pulmonary vein (LIPV) was shown by Wakili et al. as greater RF energy is required but it is also associated with oesophageal injury (Wakili et al., 2016). Another possible complication includes phrenic nerve palsy, which was reported in only one case and occurred despite prophylactic phrenic nerve stimulation (Arroja and Zimmermann, 2015).

In terms of oesophageal complications following ablation, Halbfass et al. recently conducted a retrospective study into their incidences in nMARQ vs. conventional ablation. A total 150 endoscopically detected oesophageal lesions were detected. Of these 26 occurred in 149 patients undergoing nMARQ (17.4%) and 124 occurred in 683 patients undergoing ablation using single-tip catheters (18.2%). Of the 150 endoscopically detected oesophageal lesions detected 98 were erosion injuries and 52 were ulcers of which 5 (9.6%) progressed to perforation (Halbfass et al., 2017).

### Periprocedural mortality in nMARQ vs. conventional ablation techniques

The published studies on nMARQ have demonstrated non-statistically significant higher mortality rates when compared to conventional ablation techniques (Mahida et al., 2015; Vurma et al., 2016). Of the four deaths that occurred in the nMARQ group, three were due to atrio-oesophageal fistulation and the one due to sepsis. The only multi-center study was halted immediately after the two fatalities were observed (Vurma et al., 2016). Since then it has been recognized that lower power settings were associated with less oesophageal damage (Dekker, 2016). It is possible that deaths could be prevented with lower power settings and greater operator experience. The overall mortality in the nMARQ group was 4.4% compared to 0% in the conventional group. This may be due to different sample sizes in the respective groups (1121 vs. 196). However, a multi-center survey showed that mortality was 0.1% in a sample size of 32,569 patients. This incidence remains much lower than that reported in our meta-analytical study for the nMARQ group (Cappato et al., 2009). Further studies on the efficacy of nMARQ have been stopped due to concerns of increased mortality with its use.

## Limitations

Several limitations of this study should be noted. Firstly, a high degree of heterogeneity was found in our meta-analysis, which may suggest that inconsistency of evidence and therefore our results must be interpreted with caution. This high degree of heterogeneity may be due to differences in the baseline population characteristics between the groups, such as age. Other potential contributing factors include the difference in the proportion of patients with paroxysmal and persistent AF and procedural times in each study. Secondly, cumulative analysis for parameters such as mean LVEF was not calculated due to the lack of information provided by the respective studies. There is also no data with regard to the inflammatory pathways and epigenetic modifications that were reported in the included studies and these can have an influence on therapeutic ablation response (Sardu et al., 2015, 2017).

## Conclusion

AF recurrence rates are comparable between nMARQ and conventional ablation techniques. Although general complication rates are similar for both groups, the higher mortality with nMARQ suggests that conventional techniques should be used for resistant AF until improved safety profiles of nMARQ can be demonstrated.

## Author contribution

KL and MD: data extraction and analysis, drafted manuscript; MG: data interpretation, quality analysis; TL: supervision of study, data interpretation, critical revision of manuscript; GT: supervision of study, data analysis, creation of figures, drafted and critical revision of manuscript. All other authors data interpretation, critical revision of manuscript.

### Conflict of interest statement

The authors declare that the research was conducted in the absence of any commercial or financial relationships that could be construed as a potential conflict of interest.
