# Supplementary material for: Atrial Fibrillation Recurrence and Peri-Procedural Complication Rates in nMARQ vs. Conventional Ablation Techniques: A Systematic Review and Meta-Analysis
Source: Front Physiol. 2018 May 22;9:544. doi: 10.3389/fphys.2018.00544 (PMC5985711; doi:10.3389/fphys.2018.00544)
Supplement: Supplementary file 1 [file Table_1.docx]

**Supplementary Table 1**. NOS risk of bias scale for included cohort studies

|  |  | Selection |  |  |  |  | Outcome |  |  |
| --- | --- | --- | --- | --- | --- | --- | --- | --- | --- |
| Studies | Representativeness of the exposed cohort | Selection of the non-exposed cohort | Ascertainment of exposure | Outcome of interest not present at start of study | Comparability | Assessment of outcome | Adequacy of duration of follow-up | Adequacy of completeness of follow-up | Total score  (0-9) |
| Burri 2016 | 1 | 0 | 1 | 1 | 0 | 1 | 1 | 1 | 6 |
| Dello-Russo 2015 | 1 | 0 | 1 | 1 | 0 | 1 | 1 | 1 | 6 |
| Farkash 2015 | 1 | 0 | 1 | 1 | 1 (age) | 1 | 1 | 1 | 7 |
| Laish-Farkash 2016 | 1 | 0 | 1 | 1 | 2 (age, LVEF) | 1 | 1 | 1 | 8 |
| Lauschke 2016 | 1 | 0 | 1 | 1 | 2 (age, LVEF) | 1 | 1 | 1 | 8 |
| Mahida 2015 | 1 | 0 | 1 | 1 | 0 | 1 | 1 | 1 | 6 |
| Rodriguez-Entem 2016 | 1 | 0 | 1 | 1 | 0 | 1 | 1 | 1 | 6 |
| Rosso 2016 | 1 | 0 | 1 | 1 | 1 (age) | 1 | 1 | 1 | 7 |
| Scaglione 2014 | 1 | 0 | 1 | 1 | 0 | 1 | 1 | 1 | 6 |
| Vurma 2016 | 1 | 0 | 1 | 1 | 1 (age) | 1 | 1 | 1 | 7 |
| Wakili 2016 | 1 | 0 | 1 | 1 | 2 (age, LVEF) | 1 | 1 | 1 | 8 |
| Zellerhoff 2014 | 1 | 0 | 1 | 1 | 0 | 1 | 1 | 1 | 6 |

LVEF, left ventricular ejection fraction.
